# A bibliometric review and visualization of research on myocardial ischemia/reperfusion injury in patients with diabetes mellitus from 2004 to 2024

**DOI:** 10.1097/MD.0000000000042707

**Published:** 2025-05-30

**Authors:** Yunzhao Yang, Juan Dou, Yang Wu

**Affiliations:** a Department of Anesthesiology, Renmin Hospital of Wuhan University, Wuhan, China; b Sterilization and Supply Center, Renmin Hospital of Wuhan University, Wuhan, China.

**Keywords:** bibliometric analysis, CiteSpace, diabetic myocardial ischemia/reperfusion injury, visual analysis, VOSviewer, Web of Science

## Abstract

**Background:**

The incidence of diabetes mellitus (DM) is increasing annually, and hearts of patients with DM are more sensitive to myocardial ischemia/reperfusion (MI/R) injury. However, the specific mechanisms remain unknown. Through a bibliometric analysis of published research, this study primarily summarizes and clarifies the current status of research on diabetic MI/R injury.

**Methods:**

Publications related to MI/R injury in DM from January 1, 2004, to July 31, 2024, were obtained by searching the Web of Science Core Collection. The bibliometric method was adopted using Excel 2021, CiteSpace, VOSviewer, Pajek and the R package “Bibliometrix.”

**Results:**

In total, 634 research papers and 221 reviews were published during this period that fit the criteria. The number of annual scientific publications and citations on diabetic MI/R injury has steadily increased. The majority of these studies were published by 1164 institutions from 70 countries, with China and the United States ranking first and second, respectively, in terms of publication volume. Among the leading institutions in this field, the Air Force Military Medical University, Wuhan University, and the University of Hong Kong were found to occupy the top positions. Keyword analysis reveals that recent research hotspots include antidiabetic agents, anesthetics and extracellular vesicles. Emerging trends such as ferroptosis, mitophagy, and gut microbiota are closely related to future research directions.

**Conclusion:**

This study provided a detailed overview of the key contributors, institutions, and countries, highlighting the changes and progression of hotspots in diabetic MI/R injury. China leads the world in paper production; however, international cooperation is essential for enhancing the quality of research. In our study, we found that bibliometric analysis and literature visualization will help investigators gain a deeper understanding of the progress in the research into diabetic MI/R injury.

## 1. Introduction

Diabetes mellitus (DM) is a significant global health challenge, with projections suggesting that by 2045, the worldwide prevalence of this condition will reach approximately 700 million individuals across both developed and developing nations.^[1–3]^ Notably, cardiovascular disease, particularly ischemic heart disease, emerges as the leading cause of death among individuals with DM.^[4–6]^ Although reperfusion treatments have saved many patients’ lives, DM increases susceptibility to myocardial ischemia/reperfusion (MI/R) injury, resulting in a high mortality rate among individuals with DM undergoing reperfusion therapy.^[7–9]^ Alleviating the vulnerability of diabetic myocardium to reperfusion injury is crucial for improving clinical outcomes. Notably, strict glycemic control has demonstrated limited efficacy in reducing cardiovascular mortality in patients with DM.^[4]^

Despite considerable research efforts exploring therapeutic strategies for diabetic MI/R injury, there is an urgent need to elucidate the molecular mechanisms underlying DM-aggravated MI/R pathology and develop optimized treatment protocols. Furthermore, few studies have comprehensively assessed the global research landscape in this field through systematic analysis.

Bibliometric analysis utilizes contemporary techniques that analyze countries, institutions, authors, journals, documents, and keywords to uncover knowledge structure and emerging trends within a research domain.^[7]^ To our knowledge, scholars have not conducted a systematic analysis of diabetic MI/R injury using bibliometric methods. Given the close link between DM and cardiovascular disease, this study implemented bibliometric visualization tools to systematically evaluate publication trends in research on diabetic MI/R injury. Through this approach, we aimed to evaluate the status of current focal points and emerging research trends, and provides directions for future studies on diabetic MI/R injury.

## 2. Materials and methods

### 2.1. Data collection

**Figure 1.**
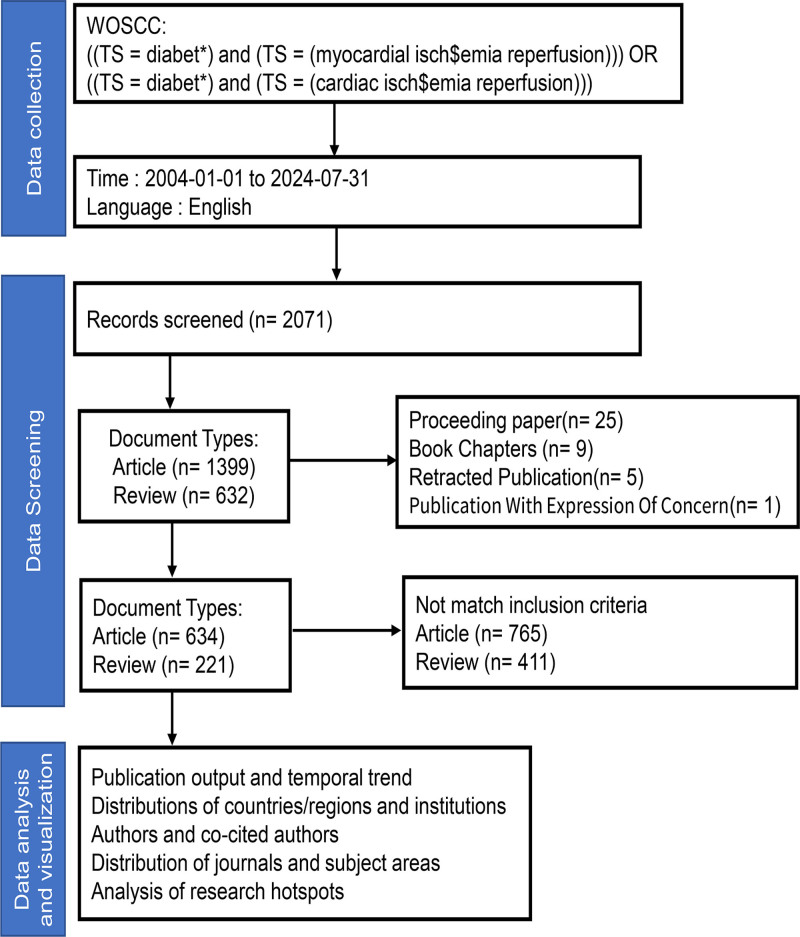
Flow chart of literature selection.

The Web of Science (WoS) Core Collection database is an extensive platform that employs a strict evaluation and selection process to determine the literature to be included, thereby guaranteeing high academic quality and reliability. It has been widely used in bibliometric studies. Our search strategy included the following terms: ((TS = diabet*) and (TS = (myocardial isch$emia reperfusion))) OR ((TS = diabet*) and (TS = (cardiac isch$emia reperfusion))). The search for publication dates was set from January 1, 2004 to July 31, 2024, based on the WoS field tag DOP. All retrieved documents were in English, based on the WoS field tag LA. In addition, articles and reviews are the types of documents included. Based on the search strategy adopted, 2071 articles were obtained from the WOS Core Collection. Of these,1399 were articles and 632 were reviews (including 25 Proceedings Papers, 5 Retracted Publication,1 Publication with an Expression of Concern, and 9 Book Chapters) (Fig. 1).

### 2.2. Inclusion and exclusion criteria for literature

After obtaining the retrieved results, studies that did not meet the inclusion criteria were manually excluded based on their titles and abstracts. Our specific inclusion and exclusion criteria were: (1) Studies that focused on diabetic MI/R injury were included, whereas those that did not clearly indicate their relevance to diabetic MI/R injury were excluded. (2) Articles and reviews published in English were also considered. (3) Studies published between January 1, 2004, and July 31, 2024. To ensure consistency of the database, data retrieval and collection were independently performed by 2 authors. The included articles were then reviewed to remove duplicates. The search yielded 855 articles of interest.

### 2.3. Data analysis

The documents were downloaded from the WOS Core Collection database as Export Records to plain text files by selecting the “full records with cited references” option. Initially, documents were imported into the CiteSpace software, where duplicate entries were identified. If there were no duplicate literature in the documents, comprehensive information (e.g., titles, authors, publication names, institutions, keywords, abstracts, publication years, and references) for each paper was extracted.^[10]^ In total, we conducted an analysis of the search results utilizing Microsoft Excel 2021, Pajek, the R package “Bibliometrix,” VOSviewer, and CiteSpace. CiteSpace (version 6.2.R6 Advanced) enabled a visual exploration of the distribution of countries, institutions, subject areas, keywords, keyword timelines, and references from the downloaded data. VOSviewer (version 1.6.20) was used to visualize the distribution of the authors, institutions, and countries.

## 3. Results

### 3.1. Analysis of the publication output in the research of diabetic MI/R injury

Acute myocardial infarction-related mortality is higher among patients with DM than those without DM.^[8,11]^ A more accurate statement would be, “Diabetes is a cardiovascular disease.” A publication period of approximately 20 years was selected to ensure that the analysis was current and relevant.

**Figure 2.**
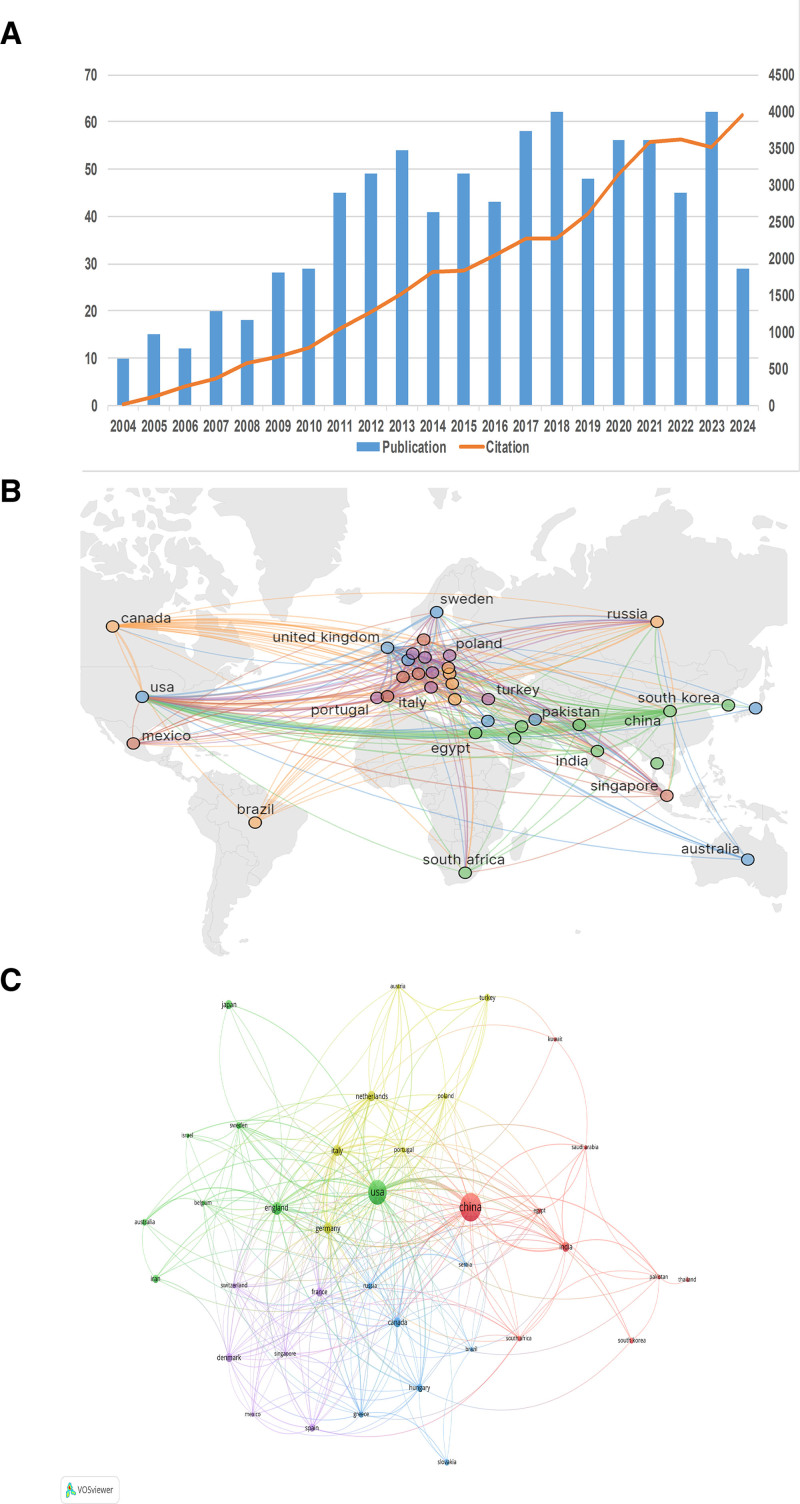
The cooperation relationship of countries/regions that published studies on diabetic MI/R injury from 2004 to 2024. (A) Publication and citation trends on diabetic MI/R injury from 2004 to 2024. Data for 2024 remain incomplete. (B) Map of cooperation among countries/regions. (C) Co-occurrence analysis of countries/regions with more than 5 publications on diabetic MI/R injury. MI/R = myocardial ischemia/reperfusion.

As shown in Figure 2A, the annual number of published articles varied from 10 to 62. In this field, the number of research papers has increased over the past 20 years. The annual number of articles peaked at 62 in 2018, and the frequency of citations reached its highest level in 2024.

### 3.2. Distributions of countries/regions in the research of diabetic MI/R injury

To date, 70 countries/regions have conducted studies on diabetic MI/R injury. In Table 1, the top ten countries are ranked according to the number of publications and citation counts. In terms of publication volume, China (268 papers) has overtaken the United States (209 papers) since 2021, followed by England (54 papers), Germany (44 papers), and Italy (43 papers). Forty percent of the of publications in the field come from the top 2 countries. Citation statistics indicate that papers published in the United States are cited the most (12,097), followed by those published in China (10,269) and England (4507).

**Table 1:** Top 10 productive countries/regions in the field of diabetic MI/R injury.

Rank	Country/region	Publications	Citations	Centrality
1	China	268	10269	0.08
2	United States	209	12097	0.23
3	England	54	4507	0.32
4	Germany	44	1974	0.04
5	Italy	43	2160	0.08
6	Netherlands	37	1607	0.04
7	Canada	34	2973	0.62
8	India	34	1433	0.01
9	Japan	29	1065	0.01
10	Denmark	28	1582	0.06

MI/R = myocardial ischemia/reperfusion.

A visual analysis of the 37 countries/regions with more than 5 papers published is shown in Figure 2B and C. Depending on the level of collaboration, these countries/regions can be categorized into approximately 5 clusters. Notably, cooperation is prominent between the United States and China, which have communicated with 34 and 30 countries/regions, respectively, creating a notable collaborative network in this field.

### 3.3. Distributions of institutions in the research of diabetic MI/R injury

**Figure 3.**
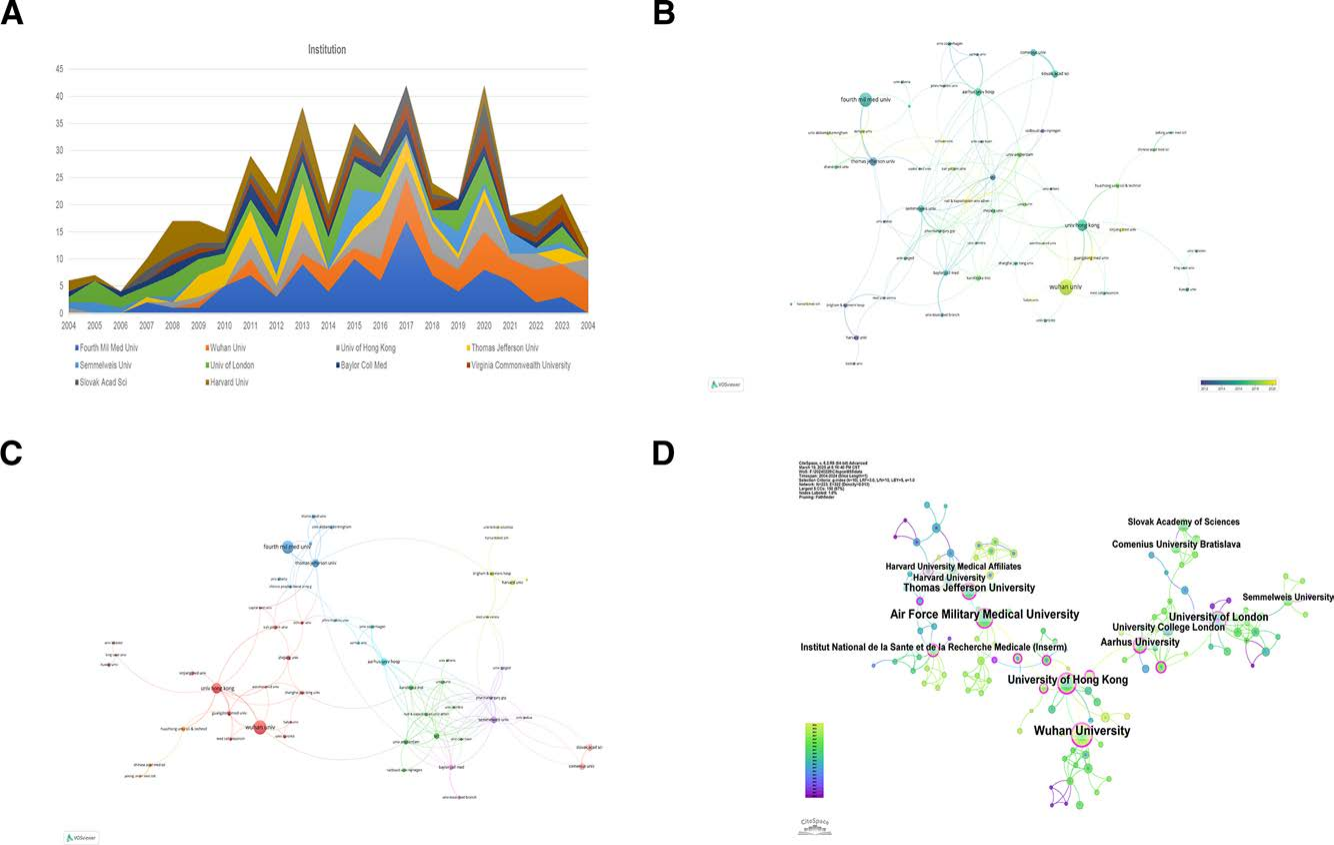
Distribution of institutions engaged in the study of diabetic MI/R injury. (A) The top 10 institutions by annual published volume. (B) Time trend of the cooperation network among institutions. (C) Network map of co-occurrence between institutions with more than 5 publications. (D) The collaborative network of institutions. The thickness of the connected lines indicates the strength of the relationship between the nodes. MI/R = myocardial ischemia/reperfusion.

In total, 1164 institutions were engaged in research on diabetic MI/R injury. We conducted statistical analysis and visual representation of 60 institutions with more than 5 publications. The institutions with the highest number of publications are ranked in Table 2, with the top 3 being from China. According to Figure 3A and B, American and European universities, such as Harvard University, University of London, and University College London, began research earlier with relatively stable publication volumes, whereas Chinese institutions, such as the Air Force Military Medical University (Fourth Mil Med Univ), Wuhan University and University of Hong Kong have experienced significant growth over the past decade. In Table 2, only the Institut National de la Sante et de la Recherche Medicale (INSERM), Air Force Military Medical University, and University of London exhibit centrality values exceeding 0.20. Wuhan University and the University of Hong Kong have published 36 and 23 articles, respectively, yet their centralities remain low, indicating a comparatively lower centrality.

**Table 2:** Top 10 institutions in terms of the number of articles published.

Rank	Institution	Publications	Citations	Centrality	Original country
1	Air Force Military Medical University	36	4442	0.27	China
2	Wuhan University	36	1968	0.02	China
3	University of Hong Kong	23	2091	0.07	China
4	University of London	21	2238	0.23	England
5	Aarhus University	18	2624	0.03	Denmark
6	Thomas Jefferson University	18	2729	0.08	United States
7	Comenius University Bratislava	14	1386	0.03	Hungary
8	University College London	14	1112	0.02	England
9	Institut National de la Sante et de la Recherche Medicale (Inserm)	13	446	0.21	France
10	Harvard University	12	1710	0.06	United States

As shown in Figure 3C, 60 institutions are categorized into 6 groups based on their cooperation. There are 2 academic groups, one represented by the Air Force Military Medical University and Thomas Jefferson University, and the other by Wuhan University and the University of Hong Kong (Fig. 3C). Similarly, in CiteSpace, institutions with a substantial volume of publications exhibit close connections with other academic institutions. Institutions with high publication output and purple circles in the network included INSERM, Thomas Jefferson University, Wuhan University, University of Hong Kong, University of London, Air Force Military Medical University, and Aarhus University (Fig. 3D).

### 3.4. Analysis of the authors in the research of diabetic MI/R injury

**Figure 4.**
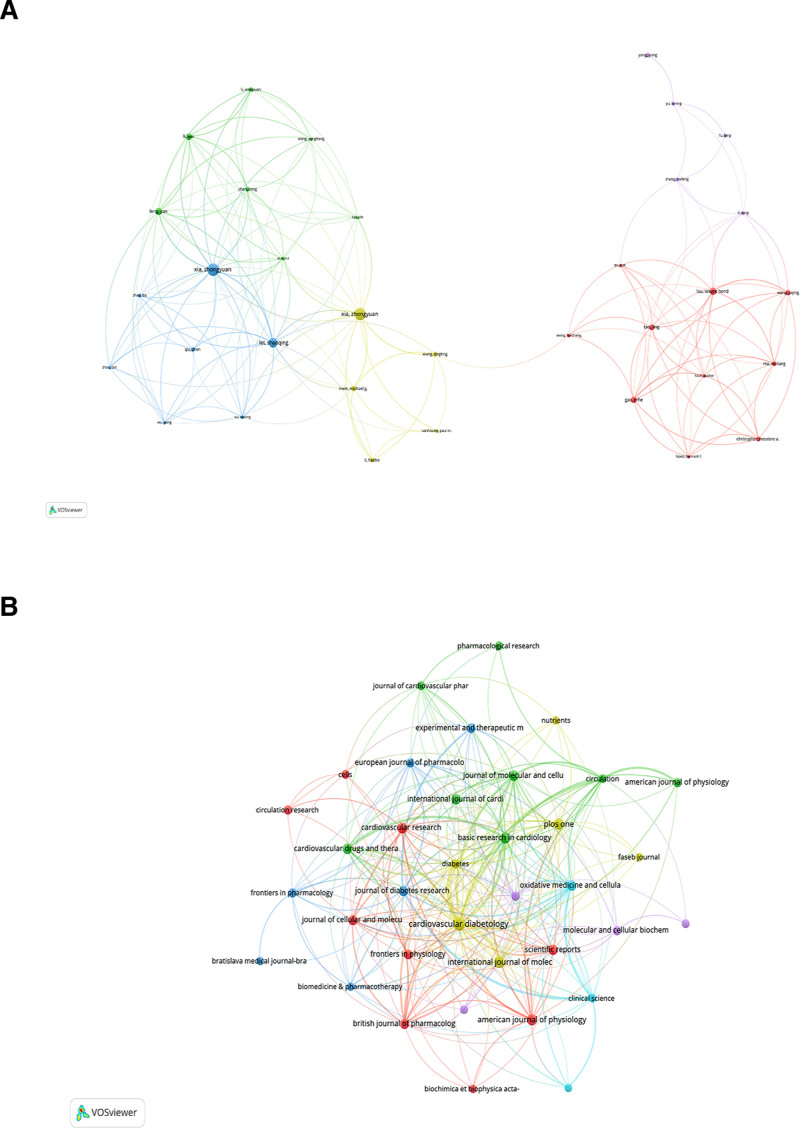
Visual analysis of the distribution of authors and journals involving diabetic MI/R injury. (A) Network visualization of collaboration among authors involved in diabetic MI/R injury. (B) Network visualization of the relationship between co-cited journals. MI/R = myocardial ischemia/reperfusion.

Data showed 4615 authors were involved in studies on diabetic MI/R injury, including 61 authors who published more than 5 articles (Fig. 4A). Most of the top 10 authors, based on publication counts, were from China and the United States (Table 3). Xia, Zhengyuan had 26 publications from Wuhan University, followed by Xia, Zhongyuan, Lei, Shaoqing, and Leng, Yan who have 25, 19 and 13 publications, respectively. Xia, Zhengyuan, the leading author of publications in this field, has conducted in-depth research in this area. VOSviewer was used to display the collaboration between diabetic MI/R injury-related authors. Figure 4A illustrates the collaboration among the authors, categorizing the coauthors into 5 distinct groups, with each color representing a specific group.

**Table 3:** Top 10 productive authors in the field of diabetic MI/R injury.

Rank	Author	Publication	Countries/ regions	Institution
1	Xia, Zhengyuan	26	China	University of Hong Kong
2	Xia, Zhongyuan	25	China	Wuhan University
3	Lei, Shaoqing	19	China	Wuhan University
4	Leng, Yan	13	China	Wuhan University
5	Lau, Wayne Bond	13	USA	Thomas Jefferson University
6	Botker, Hans Erik	13	USA	Aarhus University
7	Li, Wei	12	China	Wuhan University
8	Gao, Erhe	11	USA	Temple University
9	Hausenloy, Derek J	11	Singapore	Duke-NUS Medical School
10	Tao, Ling	10	China	Air Force Military Medical University

MI/R = myocardial ischemia/reperfusion.

### 3.5. Distribution of journals in the research of diabetic MI/R injury

Cardiovasc Diabetol (IF 8.5) and International Journal of Molecular Sciences (IF 4.9) were the 2 most-published journals (Table 4). Among the top 10 journals in terms of publication volume, 4 were in Quartile 1 (Q1) and 4 in Quartile 2 (Q2). These journals are highly recognized in the field of diabetic MI/R injury. Researchers in the fields of DM and cardiovascular research have frequently published their studies in prominent journals, reflecting the interdisciplinary nature of diabetic MI/R injury. Figure 4B shows 6 clusters of journals based on their citation frequencies. The research directions of journal articles in the same cluster were generally similar.

**Table 4:** Top 10 journals in terms of number of issues.

Rank	Journal	Publications	% of 855	Impact factor	JCR quatile
1	Cardiovasc Diabetol	29	3.39	8.5	Q1
2	Int J Mol Sci	21	2.46	4.9	Q2
3	PLoS One	19	2.22	3.7	Q3
4	Am J Physiol Heart Circ Physiol	16	1.87	4.8	Q2
5	Basic Res Cardiol	14	1.64	9.5	Q1
6	Cardiovasc Res	14	1.64	10.9	Q1
7	Cardiovasc Drugs Ther	13	1.52	3.4	Q3
8	Sci Rep	13	1.52	9.5	Q1
9	Int J Cardiol	13	1.52	3.2	Q2
10	J Mol Cell Cardiol	12	1.40	5.0	Q2

### 3.6. Distribution of subject areas in the research of diabetic MI/R injury

**Figure 5.**
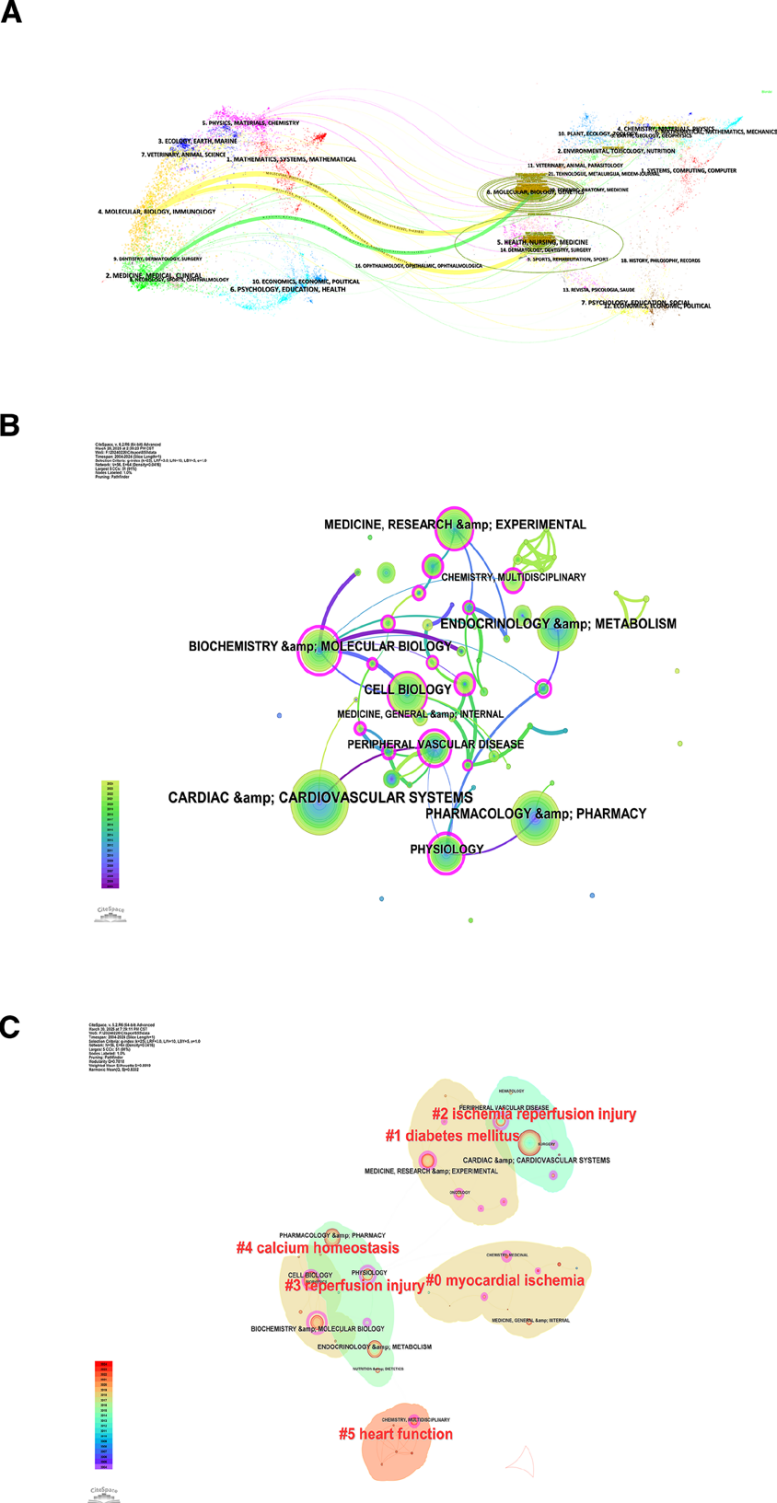
Journal analysis in the research field of diabetic MI/R injury. (A) The dual-map overlay of journals in diabetic MI/R injury. (Left: the citing journals; Right: the cited journals). (B) The citation relationships among the subject areas of the published literature. (C) Cluster view of literature identified according to subject areas. MI/R = myocardial ischemia/reperfusion.

Figure 5A illustrates the subject distribution in diabetic MI/R injury research based on a dual-map overlay. The yellow paths show that the diabetic MI/R injury papers are published in the fields of “4. Molecular Biology, Biology, and Immunology” mainly reference publications in “5. Health, Nursing, and Medicine” and “8. Molecular Biology, Biology, and Genetics.” The green path indicates that the literature published in the fields: “2. Medicine, Medical, Clinical” mainly reference publications in “8. Molecular Biology, Biology, and Genetics.” Current research on diabetic MI/R injury predominantly focuses on molecular biology with the aim of translating these findings into clinical practice.

Subsequently, the citation relationships between subject areas were analyzed using CiteSpace. According to Figure 5B, the topic of “CARDIAC & CARDIOVASCULAR SYSTEMS” has the highest number of citations. A purple circle highlights the subject areas, including “PHYSIOLOGY,” “CELL BIOLOGY,” “MEDICINE, RESEARCH & EXPERIMENTAL,” “CHEMISTRY, MULTIDISCIPLINARY,” “BIOCHEMISTRY & MOLECULAR BIOLOGY,” and “PERIPHERAL VASCULAR DISEASE.” The study of diabetic MI/R injury requires interdisciplinary integration of knowledge in the above fields. Ten literature clusters were identified based on subject areas (Fig. 5C). Clusters #0, #1, and #2 warrant particular attention. Cluster #0 includes the subject areas “MEDICINE, GENERAL & INTERNAL,” “CHEMISTRY, MEDICINAL,” and “FOOD SCIENCCE &TECHNOLOGY,” with the keyword myocardial ischemia as the central theme; Cluster #1 encompasses “MEDICINE, RESEARCH & EXPERIMENTAL,” “PHYSIOLOGY,” “SURGERY” and “IMMUNOLOGY,” with DM as the central theme; Cluster #2 encompasses “CARDIAC & CARDIOVASCULAR SYSTEMS,” “PERIPHERAL VASCULAR DISEASE,” and “SURGERY,” with ischemia reperfusion injury as the key focus. Research within these clusters has primarily focused on DM and cardiovascular disease.

### 3.7. Highly co-cited references analysis

**Figure 6.**
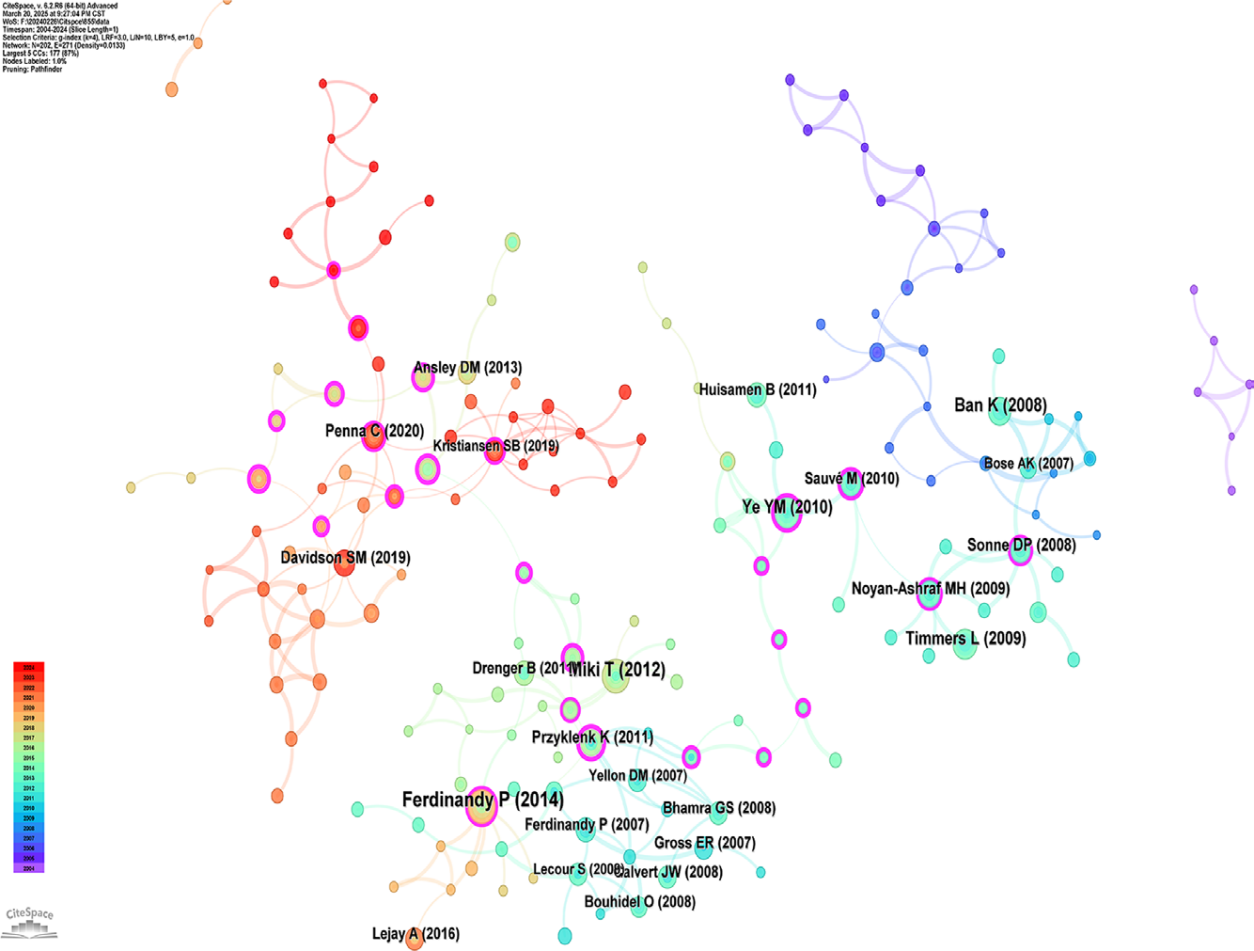
The knowledge map of co-citation reference in the field of diabetic MI/R injury. MI/R = myocardial ischemia/reperfusion.

Citation analysis reflects the importance of publications.^[12,13]^ We utilized CiteSpace to conduct a visual analysis of the co-cited references in the articles, as illustrated in Figure 6. The citation frequency is represented by the size of the nodes, while the thickness of the lines connecting the nodes indicates the strength of the relationship.^[13]^ Simultaneously, we compiled a list of the top 10 most highly co-cited publications in Table 5 to identify the core research contributions in this field. The top 10 co-cited articles were predominantly published in journals specializing in DM or cardiovascular diseases. These studies investigated the influence of DM on the cardioprotective effects of preconditioning and postconditioning. Additionally, antidiabetic medications may influence these protective mechanisms by modulating cellular signaling. Glucagon-like peptide-1 (GLP-1) has received significant attention because GLP-1 receptor agonists activate prosurvival pathways in the diabetic heart, resulting in improved outcomes after MI/R injury.^[14]^

**Table 5:** Top 10 highly co-cited references.

Rank	Article title	Authors	Journal	Year	DOI
1	Effects of diabetes on myocardial infarct size and cardioprotection by preconditioning and postconditioning	Miki T et al.	Cardiovasc Diabetol	2012	10.1186/1475-2840-11-67107.739938
2	Interaction of cardiovascular risk factors with myocardial ischemia/reperfusion injury, preconditioning, and postconditioning	Ferdinandy P et al.	Pharmacol Rev	2014	10.1124/pr.113.008300
3	Cardioprotective and Vasodilatory Actions of Glucagon-Like Peptide-1 Receptor Are Mediated Through Both Glucagon-Like Peptide-1 Receptor–Dependent and–Independent Pathways	Ban K et al.	Circulation	2008	10.1161/CIRCULATIONAHA.107.739938
4	GLP-1R agonist liraglutide activates cytoprotective pathways and improves outcomes after experimental myocardial infarction in mice	Noyan-Ashraf MH et al.	Diabetes	2009	10.1161/01.CIR.0000121747.71054.79
5	Sustained benefits in vascular function through flavanol-containing cocoa in medicated diabetic patients a double-masked, randomized, controlled trial	Timmers L et al.	J Am Coll Cardiol	2009	10.1016/j.jacc.2008.10.033
6	The myocardial infarct size-limiting effect of sitagliptin is PKA-dependent, whereas the protective effect of pioglitazone is partially dependent on PKA	Ye YM et al.	Am J Physiol Heart Circ Physiol	2010	10.1152/ajpheart.00867.2009
7	Oxidative stress and myocardial injury in the diabetic heart	Ansley DM et al.	J Pathol	2013	10.1002/path.4113
8	Cardioprotection with Cardioprotection with Postconditioning: Loss of Efficacy in Murine Models of Type-2 and Type-1 Diabetes	Przyklenk K et al.	Antioxid Redox Signal	2011	10.1089/ars.2010.3343
9	Multitarget Strategies to Reduce Myocardial Ischemia/Reperfusion Injury: JACC Review Topic of the Week	Davidson SM et al.	J Am Coll Cardiol	2019	10.1016/j.jacc.2018.09.086
10	Effect of hyperglycemia and diabetes on acute myocardial ischemia-reperfusion injury and cardioprotection by ischemic conditioning protocols	Penna C et al.	Br J Pharmacol	2020	10.1111/bph.14993

### 3.8. Keywords analysis of diabetic MI/R injury

**Figure 7.**
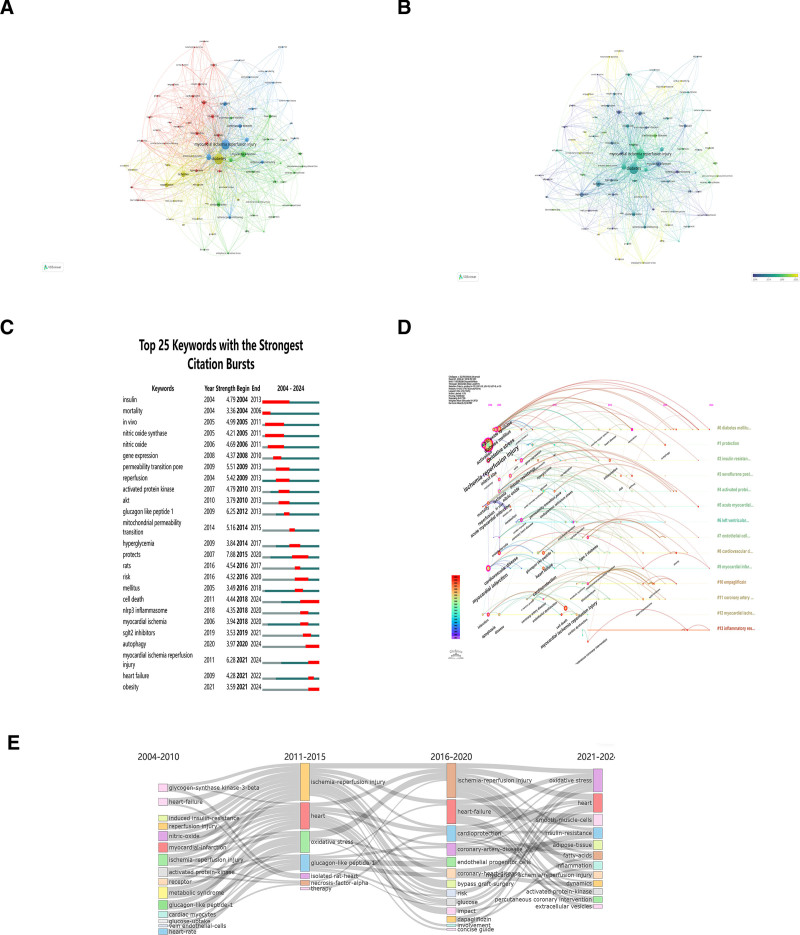
Visualization of keyword analysis. (A) Keyword co-occurrence network relationship diagram in the field of diabetic MI/R injury. (B) VOSviewer overlay visualization of keywords related to diabetic MI/R injury. The color indicates the average time; items closer to blue appeared earlier. (C) Top 25 keywords with the strongest citation frequency burst of diabetic MI/R injury. (D) Visualization map of timeline viewer related to diabetic MI/R injury. (E) Map of thematic evolution of keywords in the field of diabetic MI/R injury. MI/R = myocardial ischemia/reperfusion.

Research themes are often reflected in keywords, and we use keywords to identify research hotspots in diabetic MI/R injury.^[15]^ In VOSviewer, a threshold of 5 was applied, leading to the inclusion of 79 keywords for analysis. The top 5 keywords were “diabetes” (252 times), “myocardial ischemia reperfusion injury” (226 times), “cardioprotection” (77 times), “ischemia” (71 times), and “myocardial infarction” (65 times) as shown in Table 6. The 79 keywords were categorized into 4 distinct clusters (Fig. 7A): (1) The red cluster encompassed keywords associated with both physiological and pathological mechanisms, particularly insulin resistance, reactive oxygen species (ROS), metabolism, and hyperglycemia. (2) The green cluster primarily focuses on keywords related to cardiovascular complications and prognostic outcomes in DM, specifically coronary artery disease, atherosclerosis, heart failure, and mortality. (3) The blue cluster comprised keywords related to cardioprotective mechanisms, including cardioprotection, ischemic postconditioning and ischemic preconditioning. (4) The yellow cluster is characterized by endothelial function, particularly endothelial dysfunction, nitric oxide, and nitric oxide synthase.

**Table 6:** Top 20 keywords in the field of diabetic MI/R injury.

Rank	Keyword	Occurrences	Total link strength	Rank	Keyword	Occurrences	Total link strength
1	Diabetes	252	555	11	Heart	38	102
2	Myocardial ischemia reperfusion injury	226	468	12	Type-2 diabetes	37	77
3	Cardioprotection	77	187	13	Infarct size	31	77
4	Ischemia	71	200	14	Ischemic postconditioning	27	61
5	Myocardial infarction	65	148	15	Mitochondria	25	63
6	Reperfusion	50	141	16	Heart failure	25	55
7	Apoptosis	43	107	17	Ischemic postconditioning	24	66
8	Inflammation	42	101	18	Reactive oxygen species	22	53
9	Oxidative stress	40	92	19	Autophagy	21	63
10	Cardiovascular diseases	40	69	20	Hyperglycemia	20	45

MI/R = myocardial ischemia/reperfusion.

Figure 7B presents a time series of keywords related to diabetic MI/R injury. Notably, keywords such as endoplasmic reticulum stress (2019.38), mitochondrial dynamics (2019.8), cardiac remodeling (2020.50), nlrp3 inflammasome (2020.14), extracellular vesicles (EV) (2021.50) and ferroptosis (2022.57) have gained increasing prominence in recent years, highlighting their emerging significance in diabetic MI/R injury. Considering the unique pathological characteristics of DM, numerous studies have focused on the protective effects of antidiabetic medications. Studies on sodium glucose cotransporter-2 (SGLT2) inhibitors were published in the year 2020.75. Additionally, because patients with DM often require surgery, the role of anesthetic agents such as dexmedetomidine (2018.67) and propofol (2017.57) has also drawn attention.

Keyword bursts serve as predictive indicators for emerging research trends and can unveil potential hotspots within the research field.^[16,17]^ As shown in Figure 7C, the keyword with high citation bursts in diabetic MI/R injury is “insulin,” “mortality,” “in vivo,” “nitric oxide synthase,” “nitric oxide,” “Akt,” “activated protein kinase,” “myocardial ischemia reperfusion injury,” “reperfusion,” “GLP-1,” and “cell death.” Among the top 25 keywords still in a burst state are “myocardial ischemia reperfusion injury,” “autophagy,” “obesity,” and “cell death.”

We analyzed the timeline views of keyword progression in diabetic MI/R injury using CiteSpace. From 2004 to 2010, influential keywords included “ischemia reperfusion injury,” “myocardial infarction,” “diabetes mellitus,” “oxidative stress,” “insulin resistance,” “heart failure,” “ROS,” “nitric oxide synthase,” and “infarct size” (Fig. 7D). From 2011 to 2020, the main keywords include “type 2diabetes,” “endoplasmic reticulum stress,” “cell death,” “endothelial function,” “inflammation,” “cardiac dysfunction,” and “nlrp3 inflammasome.” Additionally, the keywords that appear more frequently between 2021 and 2024 are “autophagy,” “cardiovascular diseases,” “signaling pathway,” “cardiac remodeling” and “extracellular vesicles.” Cluster #0 (diabetes mellitus) includes the keywords “myocardial infarction,” “oxidative stress,” “nitric oxide,” “reactive oxygen species,” “nitric oxide synthase,” providing historical insights into the pathogenesis of diabetic MI/R injury; Cluster #3 (sevoflurane postconditioning) initially emerged with the keyword “high glucose,” “ferroptosis,” and “inflammation,” emphasizing the role of postconditioning and anti-apoptosis. This cluster elucidates how DM eliminates the protective effects of ischemic conditioning and explores strategies to restore these benefits. Additionally, Cluster #10 (empagliflozin) focuses on “SGLT2 inhibitors,” “oxidase activation” and “cardioprotection,” focuses on antidiabetic medicines.

The thematic evolution diagram summarizes the various evolutionary associations based on the keywords. As depicted in Figure 7E, the thematic evolution diagram demonstrates the development of diabetic MI/R injury and provides an overview of the changes in research focus over time. Based on the research focus of the publications over the past 2 decades, we have divided them into 4 distinct periods. From 2004 to 2010, researchers have explored ischemia reperfusion injury, oxidative stress, nitric oxide synthase, endothelial dysfunction, mitochondrial permeability transition pores, inflammation, and GLP-1. The second period of research (2011–2015) experienced a surge in emerging keywords. The research landscape has expanded to include endoplasmic reticulum stress, adiponectin, lipid-peroxidation, signaling pathways, metabolic syndrome, miRNA and heme oxygenase-1. Oxidative stress, mitochondrial permeability transition pore, endothelial function, inflammation, and GLP-1 remained the central foci of research during this period. From 2016 to 2020, the third period focused on apoptosis, cell death, mitophagy, protein-kinase-c, nlrp3 inflammasome, mitochondrial dysfunction, mitophagy, and nlrp3 inflammasome. During the fourth period, thematic evolution continued, with themes such as SGLT2 inhibitors, autophagy, deacetylation, ferroptosis, empagliflozin, gut microbiota, and emerging EV.

## 4. Discussion

As the incidence of DM continues to increase globally, the overall incidence of cardiovascular complications increases annually. In this study, we conducted a systematic bibliometric analysis of research on diabetic MI/R injury from January 1, 2004, to July 31, 2024.

### 4.1. General distribution on the research of diabetic

Since 2011, the annual publication output on diabetic MI/R injury has exceeded 40 papers. The number of publications and their citation frequencies serve as broad indicators of progress in the field. China and the United States are the leading contributors in terms of publication volume of research on diabetic MI/R injury. This increase in publications reflects the growing emphasis on diabetic MI/R injury studies. Regarding cooperative relationships among authors, those engaged in studies on diabetic MI/R injury studies demonstrated stronger intragroup collaboration. This indicates the need for authors to strengthen international collaboration. Xia, Zhengyuan is the most-published author in diabetic MI/R injury research. His early research primarily focused on various signaling pathways implicated in diabetic MI/R injury, including PKC β/caveolin-3/Akt, HIF-1α/HO-1, Jak/STAT3, PI3K/Akt, Brg1/Nrf2/STAT3, and PTEN/PI3K/Akt. Furthermore, his research explored the therapeutic potential of various anesthetic agents, such as sevoflurane, propofol, remifentanil, and dexmedetomidine, in mitigating diabetic MI/R injury.

Table 5 lists the top 10 most highly co-cited publications. The first most co-cited article is “Effects of diabetes on myocardial infarct size and cardioprotection by preconditioning and postconditioning.” This study summarizes how DM alters myocardial responses to preconditioning and postconditioning by disrupting intracellular signaling pathways that enhance resistance to cell death.^[3]^ The second article “Interaction of cardiovascular risk factors with myocardial ischemia/reperfusion injury, preconditioning, and postconditioning” by Peter Ferdinandy et al, highlights the complex interactions between cardiovascular risk factors and MI/R injury, preconditioning, and postconditioning. It emphasizes that DM can impair the protective effects of ischemic preconditioning, increasing susceptibility to MI/R injury.^[18]^ The third most cited article, “Cardioprotective and vasodilatory actions of GLP-1 receptor are mediated through both GLP-1 receptor-dependent and -independent pathways,” shows that GLP-1 enhances coronary flow and reduces cellular damage after MI/R injury through an NO-cGMP-dependent mechanism.^[19]^

According to Table 4, journals are primarily focused on the cardiovascular system and metabolic diseases, such as Cardiovascular Diabetology, the American Journal of Physiology-Heart and Circulatory Physiology, Basic Research in Cardiology, and Cardiovascular Research. These journals are prevalent, indicating that diabetic MI/R injury involves not only the cardiovascular and endocrine systems but also encompasses basic research, highlighting its complexity. These findings further support our analysis in the “Distribution of Subject Areas” section. Cardiovascular Diabetology (IF 9.3, Q1), International Journal of Molecular Sciences (IF 5.6, Q2), and PLoS ONE (IF 3.7, Q3), are the 3 leading journals. The top 3 ranked impact factors were Cardiovascular Research, Basic Research in Cardiology, and Cardiovascular Diabetology. Further improvements can be made to the quality of research on diabetic MI/R injury.

### 4.2. Hotspots and frontiers in the research of diabetic MI/R injury

Keyword analysis is beneficial for distinguishing important topics and emerging trends in the field.^[17]^ Researchers have generated significant interest in DM, MI/R injury, cardioprotection, apoptosis, and inflammation. Anesthetic agents, various antidiabetic medications, and EV have also received significant attention. Keyword bursts serve as valuable indicators for identifying emerging research trends.^[12,20]^ Emerging trends such as autophagy, deacetylation, gut microbiota, ferroptosis, and mitophagy are closely associated with future research directions.

### 4.3. Anesthetic agents in diabetes MI/R injury

Management of diabetic patients DM and comorbid cardiovascular disease frequently requires surgical intervention, leading to an increased research focus on the effects of anesthetic agents on diabetic MI/R injury. Recent studies have extensively explored the therapeutic potential and mechanisms of various anesthetic agents, including propofol, dexmedetomidine, opioid agonists, and sevoflurane, in mitigating diabetic MI/R injury.

It is believed that propofol reduces endothelial cell damage and suppresses myocardial autophagy in diabetic MI/R injury.^[21,22]^ Furthermore, several studies have demonstrated that propofol postconditioning inhibits miR-200c-3p, upregulates AdipoR2, and activates the STAT3 signaling pathway.^[23]^ Pretreating dexmedetomidine may confer cardioprotective effects by activating Akt, inducing GSK-3β phosphorylation, and reducing endoplasmic reticulum stress-induced cardiomyocyte apoptosis.^[24–27]^ Among opioid agonists, hyperglycemia attenuates the cardioprotective effects of remifentanil. This attenuation was possibly due to exacerbated endoplasmic reticulum stress and reduced recovery of anti-apoptotic proteins.^[28,29]^ In rats with DM, sufentanil treatment alone was ineffective in reducing MI/R injury. However, the cardioprotective effects of sufentanil are restored by insulin treatment.^[30,31]^ Sevoflurane, an inhalation anesthetic, has been extensively studied. Sevoflurane loses its protective effect on myocardium affected by DM, but deferoxamine, H_2_S, CoCl_2_, and N-acetylcysteine restore this effect.^[32–34]^ Restoration is achieved by promoting mitochondrial autophagy mediated by HIF-1/BNIP3, activation of the SIRT1/Nrf2 signaling pathway, and an increase in cardiac adiponectin levels, while reducing FoxO1 and CD36 expression.^[35–38]^

### 4.4. Antidiabetic agents in diabetes MI/R injury

In recent years, the effects of various antidiabetic medications, such as SGLT2 inhibitors, GLP-1 receptor agonists, and DPP-4 inhibitors, on diabetic MI/R injury have been extensively investigated.^[39]^ Research on SGLT2 inhibitors primarily focuses on dapagliflozin, empagliflozin, and canagliflozin. Dapagliflozin mitigates hyperglycemia-induced myocyte damage by reducing oxidative stress via NADPH oxidase inhibition.^[40]^ Additionally, it stimulates endothelial nitric oxide synthase and inhibits cardiac lipid-peroxidation, thereby alleviating diabetic MI/R injury.^[41–43]^ Empagliflozin reduces mitochondrial fragmentation in cardiomyocytes, enhances mitochondrial function, and improves blood and oxygen supply to the myocardium affected by DM.^[44–46]^ Canagliflozin decreases myocardial infarct size in rats with DM by upregulating cardiac prosurvival pathways and modulating autophagy, and reducing oxidative stress.^[39,47]^

Research indicates that pre-ischemic administration of GLP-1 receptor agonists reduces myocardial infarct size. These effects are maximized when GLP-1 receptor agonists are administered in combination with insulin.^[48]^ In diabetic hearts, liraglutide enhances survival following myocardial infarction by activating prosurvival pathways.^[14]^ Additionally, exenatide increases myocardial PKA activity and reduces myocardial infarct size in db/db mice.^[49]^

DPP-4 inhibitors, such as saxagliptin and sitagliptin, provide cardioprotective effects by reducing apoptosis and necrosiss.^[50]^ Verliptin may reduce acute mortality after diabetic myocardial infarction by promoting autophagy through Beclin-1.^[51]^ Furthermore, linagliptin and exenatide not only limited infarct size in diabetic MI/R injury model but also significantly attenuated cardiac remodeling and systolic dysfunction.^[52]^

### 4.5. Extracellular vesicles in diabetes MI/R injury

EV are systemic messengers derived from cells that carry various nucleic acids and proteins, mediating communication between cells and organs.^[5,53]^ Two of the most important types of EV are exosomes and microvesicles, both derived from different biogenesis pathways.^[5]^ In patients with DM, both the number and cargo of EV undergo significant changes compared to those without DM.^[54]^ In Davidson et al ’s study, exosomes isolated from animals with DM failed to protect cardiomyocytes from injury. However, exosomes isolated from animals without DM could restore their protective effect on DM-affected myocardium.^[55]^ Furthermore, blocking the biogenesis of miR-130b-3p may prevent dysfunctional adipose tissue from communicating with the heart, thereby providing cardioprotection against DM complications.^[5]^ Diabetic MI/R injury may be alleviated with EV-targeted therapy.

### 4.6. Strengths and limitations of the study

This study has several strengths in the field of diabetes MI/R injury research. Through a systematic bibliometric analysis, we offer researchers a comprehensive understanding of the field, along with clearer insights into research hotspots and emerging trends. However, this study has some limitations. Owing to the limitations of the analysis software, only articles from the WOS Core Collection were included; some articles from other databases, such as PubMed, were excluded. Additionally, the importance of recently published articles is often underestimated because of their short publication times and limited citations. These factors affected the research results.

## 5. Conclusion

With the increasing prevalence of DM and the heightened awareness cardiovascular complications of DM, the number of publications on diabetic MI/R injury has significantly increased. Our analysis demonstrates that China and the United States are the leading countries in terms of publication volume; however, China needs to strengthen international collaboration to enhance the quality of its research. Through a visual analysis of diabetic MI/R injury research spanning from 2004 to 2024, we identified current research areas such as anesthetics, antidiabetic agents, and extracellular vesicles. Ferroptosis, mitophagy, deacetylation, and the gut microbiota are closely associated with future research directions. This bibliometric analysis provides an effective method for evaluating research focusing on MI/R injury in patients with DM.

## Author contributions

**Methodology:** Juan Dou.

**Software:** Yunzhao Yang, Juan Dou.

**Writing – original draft:** Yunzhao Yang.

**Writing – review & editing:** Yang Wu.
